# Consciousness reduced: The role of the ‘idiot’ in early evolutionary psychology

**DOI:** 10.1177/0952695120911557

**Published:** 2020-07-07

**Authors:** Simon Jarrett

**Affiliations:** 4894 Birkbeck, University of London, UK

**Keywords:** animal psychology, consciousness, evolutionary psychology, idiocy, intellectual disability

## Abstract

A conception of the idiotic mind was used to substantiate late 19th-century theories of mental evolution. A new school of animal/comparative psychologists attempted from the 1870s to demonstrate that evolution was a mental as well as a physical process. This intellectual enterprise necessitated the closure, or narrowing, of the ‘consciousness gap’ between human and animal species. A concept of a quasi-non-conscious human mind, set against conscious intention and ability in higher animals, provided an explanatory framework for the human–animal continuum and the evolution of consciousness. The article addresses a significant lacuna in the historiographies of intellectual disability, animal science, and evolutionary psychology, where the application of a conception of human idiocy to advance theories of consciousness evolution has not hitherto been explored. These ideas retain contemporary resonance in ethology and cognitive psychology, and in the theory of ‘speciesism’, outlined by Peter Singer in *Animal Liberation* (1975), which claims that equal consideration of interests is not arbitrarily restricted to members of the human species, and advocates euthanasia of intellectually disabled human infants. Speciesism remains at the core of animal rights activism today. The article also explores the influence of the idea of the semi-evolved idiot mind in late-Victorian anthropology and neuroscience. These ideas operated in a separate intellectual sphere to eugenic thought. They were (and remain) deeply influential, and were at the heart of the idea of the moral idiot or imbecile, targeted in the 1913 Mental Deficiency Act, as well as in 20th-century animal and human consciousness theory.

In 1882, George Romanes, pioneer of post-Darwinian animal psychology and inheritor of Darwin’s notes on instinct (Young, 1985: 59), recorded the details of a fascinating fight he had witnessed in the animal kingdom. The battle was riveting. One combatant was grabbing its much larger rival in an unshakeable grip, while the other tried to throw the opponent off. The large captive swung about trying to dislodge the captor, eventually fastening on to a handy nearby object, and then ‘began the most extraordinary series of movements which were obviously directed at ridding itself of the encumbrance. It dashed from side to side in all directions with a vigour and suddenness which were highly astonishing’ (Romanes, 1886[1882]: 18–19). However, the plucky smaller assailant held on with great tenacity in the face of its opponent’s manoeuvres until the trial of strength eventually ended, after several minutes of struggle, with the aggressor being thrown violently off by its intended victim (ibid.).

Just as astonishing as the fight was that Romanes was observing it through a microscope: It was taking place in a tiny water-filled dish, and involved two rotifer, microscopic pseudocoelomates, a group of tiny invertebrates, simple organisms with fluid-filled body cavities. The fight was described in the chapter on protozoa in Romanes’ 1882 book *Animal Intelligence*. Reflecting on what he had seen, he wrote,

It is difficult to believe that these little animals are not activated by some amount of intelligence…two of them having a fight – one grabbing another by its forceps, the other trying to throw itself.…The entire scene was as like intelligent action on behalf of the animals as could be imagined.…If we were to depend on appearances alone, this one observation would be sufficient to induce me to attribute conscious determination to these microscopical organisms. (Romanes, 1886[1882]: 18–19)

While not entirely convinced that there was conscious intention behind the actions of his prize-fighting animal organisms, Romanes was not prepared to fully discount their capacity for volition and intention. On the other hand, there was a type of human animal in whom he detected only the very lowest levels of consciousness, if any. These were human ‘idiots’,

a class of persons…of peculiar interest in relation to mental evolution, because in them we have a human mind arrested in its development as well as deflected in its growth…supplying to the comparative psychologist very suggestive material to study. (Romanes, 1883: 181)

During his search for intelligent habits in non-human animals, Romanes had taken the time to visit idiot asylums, where he had observed ‘tricks of manner’ (‘non-intelligent habits of a non-adaptive character’) in the inmate population, and noted that ‘the lower the idiot in the scale of idiotcy, the more pronounced is this peculiarity’ (Romanes, 1883: 181–2). In this world of high-performing microscopic rotifer and low-performing humans, it seemed that the consciousness gap between human and animal species was not as unbridgeable as many thought, and that an explanation of the evolution of consciousness and mental states might be within reach.

This article will examine the use, from the late 19th century, of a fabricated conception of the idiotic mind to overcome the explanatory challenges posed by the theory of natural selection. The purpose was to demonstrate that evolution was a mental as well as a physical process, and that consciousness had emerged somewhere on the adaptive journey from single-cell organism to highly complex human mammal. Darwin in his later works, and then the new school of comparative psychologists led by Romanes, weaved a notion of the semi-evolved idiot human into the wider intellectual shift to a reductive formulation of the human body and mind/brain. The purpose was to narrow the consciousness gap between human and non-human animals, and to demonstrate that evolution was a mental as well as a physical process. In this explanation, the idiot brain represented an important staging post on the journey from unconscious organism to thinking human, a notion that endures today.

## Historiography

The article addresses a significant lacuna in the historiographies of intellectual disability, evolutionary psychology, and the history of animal consciousness, evolution, and behaviour. In each of these fields, the late-Victorian use of the human idiot mind to advance theories of animal and human consciousness has received little focus.

Major work on the history of intellectual disability in this period has concentrated on the interplay between eugenic science and wider social and ideological factors in the formulation of ideas of mental deficiency and feeblemindedness. Thomson (1998) argues that the Mental Deficiency Act of 1913 was influenced by eugenic science and fears of degeneration, but also by a late-Victorian moral and ideological anxiety about mass democracy, responsible citizenship, and the place of those deemed naturally irresponsible and incapable. Patrick McDonagh (2008) has made the counterargument that the impulses leading to the 1913 act were predominantly eugenic, with the mentally deficient used as a contrast group to justify the extension of rights and suffrage to the majority population. David Wright (2001) attributes the proto-eugenicist underpinnings of the 1890s debate about the institutional confinement of idiots and imbeciles to the collapse of earlier therapeutic optimism and evangelical humanitarianism, and its replacement by darker forms of hereditarianism and social pessimism. Mark Jackson (2000) argues that the idea of feeblemindedness derived from medical attempts to explain conditions in the borderland between normalcy and mental dysfunction, where simplistic eugenic explanations of mental deficiency were weakest. Others have simply conflated Darwinian evolution theory with the ideology of social Darwinism (Smith, Noll, and Wehmeyer, 2013: 119). Each of these has viewed the framing of the so-called idiot mind in this period primarily through the eugenic lens, and the lesser or greater extent of its influence on social policy, theories of mind, and public opinion. Only Gelb (2008) has explicitly referenced Darwin’s use of idiocy to narrow the consciousness gap and advance evolutionary theory.

I suggest that developments in psychology at this time offered a different framing of the idiot mind, which demonstrated not the degeneration of the human mental state proposed by social Darwinism, but its progress towards perfection through the evolutionary process. The idiot mind offered a convenient proof of an intermediate staging post in the progression of mind from the reflexivity and instinctiveness of the non-human animal to the intentional consciousness of the fully formed human brain. In this sense, the idiot was not seen as a degenerative threat to civilisation, but as an object of observation and experimentation to explain the passage of the human mind from its atavistic animal origins. In this way, the idiot could be used to throw light on the delineation of the borderland between humans and non-humans. Late 19th-century psychologists, mental scientists, and social theorists, and their 20th-century successors, came to see idiots in their institutionalised settings as valuable explanatory artifices and experimental tools for their theories of animal and human mind. They placed idiots close to, or even among, non-human animals in public perception.

Closing the consciousness gap across species has endured as a central concern of ethologists, animal behaviourists, and psychologists who seek to demonstrate (or disprove) the close evolutionary connectedness of animal and human minds, and to make claims about the levels of consciousness present in non-human species. Derek Bickerton (2000: 863) has summarised the debate as ‘whether human consciousness is isomorphic with, disjoint from, or to some extent overlaps animal consciousness’. Marian Dawkins has asserted that ‘the consciousness problem persists for animal behaviourists’, and that explaining mental evolution remains a challenge to the theory of natural selection, particularly if consciousness is linked to the capacity to perform complex cognitive tasks rather than simply the ability to experience emotion (Dawkins, 1993: 7–8; 2000: 885). Theorists and philosophers of animal behaviour and consciousness frequently acknowledge their debt to Romanes (Rollin, 1989: 46–8) and his late-Victorian peers, including Conwy Lloyd Morgan, whose ‘canon’ concerning the validity of non-human consciousness claims (discussed below) remains today a central topic of debate in the field. However, while these authors devote much attention to the racialised view of consciousness among early Darwinists that placed ‘savages’ and ‘barbarians’ close to the cross-species border area (Cartmill, 2000; Kimler, 2000), they do not explore the framing of the idiot mind as the lowest, and most animal-like, form of human consciousness. Historians too have highlighted the racial linkages spawned by the search for explanations of evolutionary consciousness, but have not examined the more overt linkages made to idiotic minds. Radick has noted that in the Victorian view of a progressive and gradual chain of evolution, ‘its highest products were humans, the lowest humans were the savage and barbarian races’ (Radick, 2007: 6). However, below even these racialised caricatures, crossing the border into animality, stood the semi-human form of the idiot.

## Influence of early comparative psychology on modern scholarship and research

Curious inverted echoes of the Victorian preoccupation with the defective human as an evolutionary link to animal minds are discernible in recent and current scholarship. This is evident in the claim that we denigrate non-human species by conceptualising animal minds as ‘deformed’ in contrast to human minds:

As materialists and Darwinians, scientists are compelled to recognise our close animal relatives as being in effect deformed human beings, differing from us only in certain genetic rearrangements that make them grow up funny-looking and stupid. Since our closest relatives have brains, bodies and behaviours that correspond to our own in many ways, it seems reasonable to assume that they have minds and experiences something like ours. (Cartmill and Lofstrom, 2000: 833)

Ramsden and Wilson (2014: 203) claim that human reflections on the natural world produce ‘the seemingly deficient animal mind’.

The psychologist Stephen Pinker echoes his Victorian forbears in seeing the animal mind and the impaired human mind as key entrance points to understanding the operation of the ‘normal’ mind (along with a 21st-century interest in the robotic mind):

To appreciate what goes on in our minds when we effortlessly learn from other people, we have to imagine what it would be like to have some *other* kind of mind. Fortunately cognitive scientists have imagined it for us by plumbing the minds of robots, animals and people whose minds are impaired. (Pinker, 2002: 61; emphasis in original)

The primatologist Jane Goodall wrote in her foreword to the philosopher Bernard Rollin’s treatise on animal consciousness and pain:

As Rollin points out, the common-sense view of most people has always been that animals, like pre-verbal children and the mentally retarded, experience a variety of *human-like* feelings including pain. It was from this perspective that Darwin, for example, argued that there was continuity in the evolution of mind as well as structure. (Goodall, 1989: vi–vii; emphasis in original)

The presentation of the idiot mind as a mechanism lying somewhere between the human and other species, conveniently providing insights that elevate the consciousness of otherwise unobservable and unknowable minds, retains its force.

The post-Darwinian drive to close the human-to-animal consciousness gap retains great significance. Modern animal science continues to seek to bridge the divide by exemplifying amplified consciousness and humanlike traits in non-human species, such as rudimentary intentional language skills in the grey parrot, social awareness in monkeys, and indicators of ape consciousness (Pepperberg, 1999; Pepperberg and Lynn, 2000; Savage-Rumbaugh, Fields, and Taglialatela, 2000; Seyfarth and Cheney, 2000). Such studies have spawned a rash of popular science and psychology books that aim to demonstrate the proximity of animal and human minds, and consequently advance the case for animal rights. These range from the ethologist Frans de Waal’s *Are We Smart Enough to Know How Smart Animals Are?* (2016) to Jonathan Balcombe’s eye-wateringly anthropomorphic *What a Fish Knows: The Inner Lives of Our Underwater Cousins* (2017). Perhaps unknown to at least some of their authors, these works reflect closely the style and content of the work of Romanes and his peers over a century earlier. Marian Dawkins, an animal behaviourist and advocate of animal consciousness and rights, has warned that ‘studies of mental ability in animals are peculiarly susceptible to overinterpretation’ (Dawkins, 1993: 178).

Cartmill has pointed out how from the beginning, Darwinians advanced anthropopsychic views by exaggerating the humanlike characteristics of beasts and presenting certain types of human as ‘quasi-simian intermediates’ (Cartmill, 2000: 839). It is in the interests of those seeking to promote the idea that animals have high levels of consciousness to find human beings who appear to exist at a low level of awareness, against whom non-human animals contrast favourably. In its most extreme form today, this emerges as the modern theory of ‘speciesism’, outlined by the ethical philosopher Peter Singer in *Animal Liberation* (1975), which has at least some of its intellectual roots in late 19th-century post-Darwinian developments in psychology and philosophy. Speciesism argues that equal consideration of interests is not arbitrarily restricted to members of the human species, and that moral standards applied to human beings should extend to other animals. The corollary of this argument, according to Singer, is that the right to life of some animals is greater than that of what he calls ‘retarded’ humans, on the grounds that the animals have a greater self-awareness and capacity for meaningful relationships than the severely retarded human. This argument is used as a justification for both the preservation of animal life and the euthanasia of disabled human infants (Singer, 1975). The theory of speciesism remains at the core of animal rights activism today. I will argue that it is significantly influenced by the comparative psychology and philosophy of the immediate post-Darwinian period, and carries the same fabricated idea of the unevolved idiot mind as was used to close the human–animal consciousness gap to support evolutionary theory.

The article includes an examination of the work of the philosopher Henry Sidgwick (1838–1900), who is a major influence on Singer and his circle (Lazari-Radek and Singer, 2014) and who responded to the philosophical challenge of evolutionary psychology by developing a theory of evolved ‘desirable’ consciousness, which is clearly discernible in contemporary arguments advanced in support of the theory of speciesism. I will place evolutionary psychology’s reformulation of the idiot mind in the wider context of Sidgwick’s move to moralise the new concept of evolved consciousness, and of similar moves to moralise consciousness, using the idiot mind as a marker, by the medico-psychological specialist Henry Maudsley. All of this was influenced by concurrent neurophysiological advances in identifying and understanding cerebral localisation, and sources of intelligence in the human and animal brain, led by David Ferrier in the 1870s. I will argue that these new modes of thought about the idiot mind operated in a separate (if connected) intellectual sphere to that of degenerationist and eugenic thought. They were (and remain) deeply influential in animal psychology, science and history, consciousness theory, moral philosophy, and the ideology of animal liberation. They were also at the heart of the idea of the moral idiot or imbecile, targeted in the 1913 Mental Deficiency Act, and remain embedded, if somewhat disguised, in modern notions of intellectual disability, particularly in its relation to cognitive psychology.

## Idiocy in the concept of mental evolution

While Darwin was famously reticent about the evolution of humans in *On the Origin of Species* (1859), smuggling in his line ‘light will be thrown on the origin of man and his history’ on his final page (Darwin, 1998[1859]: 368), he would attempt to place the human species within his overarching theory in *The Descent of Man* (1871) and *The Expression of the Emotions in Man and Animals* (1872). He had predicted in *Origin*, somewhat disingenuously, that ‘in the distant future…psychology will be based on a new foundation, that of the necessary acquirement of each mental power and capacity by gradation’ (Darwin, 1998[1859]: 367–8). His theory would in fact unleash an immediate and furious public dispute about the physical and mental evolution of the human species, most notably in the heavyweight confrontation between Thomas ‘Darwin’s Bulldog’ Huxley and Bishop Samuel ‘Soapy Sam’ Wilberforce in the 1860 Oxford evolution debate, just one year after the publication of *Origin*. The decade of discussion about human evolution that followed meant that by the time Darwin approached the subject in the early 1870s, the themes and parameters of the debate had already been exhaustively placed in the public domain. This allowed him the freedom to speculate on theories of the human–animal continuum that were already widely known and enjoying a degree of acceptance, without causing a high level of shock and outrage.

In these attempts by Darwin to explain mental evolution, the human idiot was presented as a critical signifier of the human–animal link. In *Descent*, Darwin drew on Vogt’s 1867 *Memoire sur les microcéphales* to place microcephalic idiots on a scale of arrested development that linked them both to ‘lower types of mankind’ (by which he meant ‘savages’) and lower animals. He noted their deviation in appearance and brain shape from ‘normal men’. They had smaller skulls, less complex convolutions of the brain, projecting foreheads, and strongly prognathous jaws (Darwin, 2004[1871]: 54). This description mirrored the stereotypical idiotic countenance first systematised in Lavater’s *Essays on Physiognomy* (1774–8) and subsequently re-presented by the phrenological school throughout the 19th century, from Gall and Spurzheim through to Galton and Lombroso. These facial, brain, and skull characteristics united the idiot type, in the phrenological world view, with the savage type.

While linking the idiot up towards the human savage in the developmental scale, Darwin also made the downward link to lower animals. He noted idiots’ ‘extremely feeble’ mental faculties, lack of speech, inability to attend, and propensity for imitation. Idiots were also, he suggested, ‘strong and remarkably active, continually gamboling and jumping about, and making grimaces. They often ascend stairs on all-fours, and are curiously fond of climbing up furniture or trees’ (Darwin, 2004[1871]: 54).

This reminds us, he claimed, ‘how lambs and kids, originally alpine animals, delight to frisk on any hillock, however small’ (Darwin, 2004[1871]: 54.). He went on to describe a number of other ways in which idiots resembled lower animals: smelling every mouthful of food before eating it; the ape-like use of mouth in aid of hands while hunting for lice; filthy habits; lack of decency; and, to top it all, remarkably hairy bodies (ibid.).

Darwin placed the similarity of the idiot to lower animals under the category of ‘reversion’ as well as ‘arrests of development’. By ‘reversion’ he meant the way in which an arrested bodily or mental structure resembles a lower structure, even if it continues to grow. It was of interest because

the lower members in a group give us some idea how the common progenitor was probably constructed.…The simple brain of a microcephalous idiot, in as far as it resembles that of an ape, may in this sense be said to offer a case of reversion. (Darwin 2004[1871]: 55)

In reversion theory, idiots in effect offered an opportunity to observe a state of embryonic development arising from an earlier state of existence – they were an evolutionary snapshot. In this guise, they could also offer clues to the origin of language, their parrot-like imitative tendencies closely allying them to ‘monkeys…and…the barbarous races of mankind’ as a prior indicator of complex language systems (ibid.: 109).

The next year, in *Expression of the Emotions*, Darwin again presented the idiot as occupying an important bridging role in the human–animal continuum. He argued that laughter and smiling in idiots, although their ‘most prevalent and frequent of all the emotional expressions’, was frequently senseless and unrelated to any discernible cause (Darwin, 2009[1872]: 182). At other times, idiots would ‘grin, chuckle or giggle, whenever food is placed before them, or when they are caressed, are shown bright colours, or hear music’ (ibid.: 183). Darwin agreed with his main informant on idiot behaviour, James Crichton Browne, superintendent of the West Riding Lunatic Asylum in Wakefield, Yorkshire, that ‘the joyousness of most of these idiots cannot possibly be associated with any distinct ideas: they simply feel pleasure, and express it by laughter or smiles’. The argument was that the laughter of the idiot was reflexive, analogous to our inability to prevent ourselves laughing when tickled. While ‘grown-up persons’ graduate to cerebrally advanced laughter about complex incongruities and absurdities, idiots remain mired in childlike uncomprehending laughter, much like the anthropoid apes, who, Darwin claimed, ‘utter a reiterated sound, corresponding to our laughter, when they are tickled, especially under the armpits’ (ibid.: 184). Unlike laughter, Darwin was informed by Crichton Browne, blushing was rare among idiots; in fact, Browne claimed never to have seen a genuine blush on the face of an idiot. This, Darwin concluded, was because blushing ‘is the most peculiar and most human of all expressions’, caused not by physical stimuli but purely by the mind, through intellectually advanced feelings of shame or embarrassment (ibid.: 186–7).

Both of these idiotic characterisations – reflexive laughter and inability to blush – were part of a formulation of the idiot mind as at best largely instinctive and at worst largely reflexive. Those actions that could be caused entirely by physical stimuli without recourse to the brain were prevalent in idiocy, while those that required some level of mental faculty and capacity were absent. The purpose of such a formulation was to demonstrate that by applying reversion theory, we could observe in the arrested idiot mind a specific point on the journey of mental evolution from unconscious reflex of the simple organism, through partially conscious instinctive actions, to the fully conscious intentionality and volition of the fully developed human type. The idiot mind also showed uncanny resemblances to the minds of non-human animals, further proof of the interconnectedness and common origins of man and other animal species. The idiot in Darwin’s presentation was what Mitchell and Snyder have called in literature a ‘narrative prosthesis’, where the disabled person is deployed as an essential metaphorical plot device to carry a storyline (Mitchell and Snyder, 2000). In Darwin’s case, the idiot was deployed to lend scientific evidence to his drama of mental evolution, a crutch upon which his thesis could gratefully lean.

Both *Descent* and *Expression* have been characterised as problematic, not only by modern historians of the human sciences, but also by Darwin’s contemporary observers. As Roger Smith has summarised it, *Descent*

never achieved the intensity or command over its materials that made the *Origin* so persuasive a book. His argument was necessarily indirect since there was no record of human evolution. He maintained that if *Homo Sapiens* differs, for example, in intelligence, from animals by degree but not in kind, then it is plausible to believe in human evolution.…As a result, he read into animal nature what is characteristic of human nature, and he used what he found in animal nature to confirm continuity between humans and animals. (Smith, 2013: 55–6)

The silent, non-progressing and infinitely malleable idiot type was a perfect underscore to the unification of human and animal characteristics that Darwin’s enterprise required. In 1876, George Mivart, a noted sceptic about the animal consciousness claims of the new animal psychology, attacked Darwin for his ‘biological or inverted anthropomorphism which has led to that exaggerated interpretation of animal instincts, of which Mr Darwin, in his “Descent of Man” has given us…such an ever-memorable example’ (Mivart, 1876: 200). Mivart, an initial supporter of evolutionary theory, later turned against the idea that the human intellect was subject to the evolutionary process and became an implacable opponent of Darwin. However, his critique of Darwin’s over-attribution of conscious intention to animal actions captures the loss of rigour that occurred between *Origin*, and *Descent* and *Expression*. He objected to Darwin accepting anecdotes about animal behaviour, often second-hand, with little criticism (ibid.: 204–5).

The same could be said of Darwin’s speculations on idiots. As well as his vicarious consumption of Crichton Browne’s anecdotes from West Riding, all Darwin’s other observations on idiots were taken from secondary sources. Some of them were observations of just one person’s behaviour or appearance: ‘Pinel has…given a striking case of hairiness in an idiot’, read one of his footnotes (Darwin, 2004[1871]: 54). He was also influenced by existing idiocy tropes, particularly that of animal-like behaviours, which had circulated throughout the century. The medical superintendent of the Pennsylvania Training School for Feeble-Minded Children, Isaac Newton Kerlin, supplied the following anecdote in 1858, the year before *Origin*, about his ‘discovery’ of an imbecile child called Beckie:

She was in the grove alone: and carried in her little clenched fingers, a quantity of sticks and stones: her form was crouched, and she moved about among the leaves, apparently in search of something; but on our approach, she bounded away with the grace and lightness of a startled gazelle. We followed her, and after much coaxing and many manoeuvres, succeeded in getting her to approach. (Kerlin, 1858: 16)

The style and tone of this account are reminiscent of Darwin’s narratives of encounters with animals in *Voyage of the Beagle* (1839). Just as what Robert Young has called ‘Darwin’s anthropomorphic way of writing about selection’ and even his ‘rank anthropomorphism’ (Young, 1985: 92–3) created a sense of conscious intention in his descriptions of animal actions, so he drew on the inverted anthropomorphism of culturally embedded Victorian tropes about idiot animality to place animal-like humans close to humanlike animals in the evolutionary scale.

## The growth of animal psychology

The sense given by the somewhat anecdotal and speculative *Descent* and *Expression* that mental states were not the central concerns of Darwin the biologist was reinforced when he passed his notes on instinct to his young protégé George Romanes. This marked a symbolic handover from biology to psychology, with Romanes setting out ‘to do for the evolution of the mind what Darwin had done for the evolution of the body’ (Young, 1985: 58). Romanes laid out in the introduction to his *Animal Intelligence* (1882) his grand scheme to publish a series of works that would systematically examine mental evolution in both animals and man, embed comparative psychology in the hierarchy of the sciences, and apply empirical observation and experimentation (rather than anecdote and speculation) to the theory of descent. He completed *Animal Intelligence*, *Mental Evolution in Animals* (1883), and *Mental Evolution in Man* (1888), although his premature death in 1894 meant that projected work on intellect, emotions, the will, morals, and religion was not completed (Smith, 2012).

Romanes picked up enthusiastically on the potential of the idiot type to throw light on the evolution of both the animal and the human mind, although unlike Darwin he made use of his own observations from asylum visits as well as published papers and information gleaned from asylum superintendents. Romanes continued to develop the emerging thesis of the evolution of mind: that it began with reflex (entirely unconscious action of the nervous system in response to external stimuli), progressed to instinct (learned adaptive behaviour to specific stimuli passed on through whole species and containing a limited amount of consciousness), and culminated in the higher species in conscious action (intentional individual reactions to previously unencountered stimuli). Consciousness was therefore a shift from passive reflexivity to purposeful intentionality (Romanes, 1886[1882]: 4–8). The higher up the evolutionary scale a species found itself, the more conscious and less reflexive or instinctive it was.

Romanes framed the partially evolved human idiot type as an example of a largely instinctive and reflexive being. He first cleared up the fallacious assumption that brain size was important, pointing out research presented at the International Congress of Psychologists in Paris in 1878 that demonstrated that ‘idiotcy is compatible with large and apparently well-developed brains, the amount of grey matter in one instance being “enormous”’ (Romanes, 1883: 45). This was compatible, he observed, with findings from the animal kingdom where skull or brain size represented ‘but a very uncertain index of the level of intelligence which is attained by the animal’ (ibid.). Far more important than quantity was the quality of brain, and its capacity for conscious determination over its propensity to instinctive or reflexive action. Reaction times, he noted, quoting research from Italy, were faster among the non-educated than the educated, and fastest of all among idiots. This might have appeared a surprising finding, given that the idiot had a reputation for dullness, slow-wittedness and unawareness of their surroundings. For Romanes, however, this was highly significant, because ‘the time-relations observation in perception…with reference to the theory of the rise of consciousness, and also of the physiological side of mental evolution in general…[is] of the highest importance’ (ibid.: 138–9). Idiots and the mentally dull reacted quickly because they did not think; they were purely reflexive. Those with higher levels of consciousness reacted more slowly because their deliberative thought processes interfered with and overrode the reflexive response. The idiot’s transformation from a slow and lugubrious brain to a quick and responsive brain was not in their favour: It demonstrated their reversionary backwardness, illuminated the evolutionary process of the mind, and proved their reflexive mental state.

Further evidence of the idiot’s ‘peculiar interest in relation to mental evolution’ with their mind ‘arrested in its development as well as deflected in its growth’ (Romanes, 1883: 181) was furnished by Romanes’ observations of instinctive mannerisms at work in idiot asylums. He noted in particular what he called ‘the extraordinary character and variety of the meaningless tricks of manner which are everywhere being displayed’ (ibid.). These mannerisms were also often observed in human children, but would mostly disappear as the child developed (or be eradicated by supervising adults). In idiots, however, they were ‘wonderfully persistent’, despite being both ludicrous and meaningless. The lower the idiot in the scale of idiocy, the more pronounced were these peculiarities. They included continuous ‘to and fro’ or rhythmical movements, and habitual movements of the hands, limbs, and features (ibid.). Romanes compared this to similar ‘meaningless trick’ behaviours by animals. An ‘idiotic’ dog, for example, might turn around up to 20 times before lying down. There was no meaning or purpose in this action, but in Romanes’ view it was probably a secondary instinct relating to its ancestors forming a bed in long grass (ibid.: 193). The survival of these instincts was an example of a formerly intelligent and necessary action becoming habitual and eventually reflexive in the less mentally well endowed, long after its usefulness had passed. So it was with the rhythmical, inexplicable, unnecessary movements of the idiots in the asylum – a glimpse into the necessary actions of evolving ancestral humans, frozen as reflexive, useless actions in their peculiar reversionary descendants, and placing the human ever nearer to the non-human animal.

Despite his promise to bring a culture of scientific observation to the investigation of animal mental states, Romanes frequently drew heavily on anecdote and could be highly anthropomorphic in his interpretations of animal behaviour, in order to prove evolutionary continuity. Studying the ‘lower forms’ of human allowed him to claim that ‘in some respects the higher receptual life of brutes attains almost as high a level of ideation as the lower conceptual life of man’ (Romanes, 1888: 215). Often only the ability to use language divided man and beast, and in the human idiot form language (even by signs) could be entirely absent or purely imitative, like the parrot. His compression of species difference, achieved through the elevation of the consciousness levels of some types of animal and the diminution of the conscious faculties of some types of human, set a framework for animal psychology that would be influential in the conceptualisation of human and animal minds even when his tendency to anthropomorphism was questioned. Robert Young has suggested he could be considered ‘the most important pioneer in comparative psychology’ (Young, 1985: 59). His contribution to a refashioning of the idea of the idiot mind to conceive it as on a level with the animal mind, and its occupation of a signifier role in the explanatory system of evolution, was paradigmatic.

The closing of the human–animal consciousness gap required a reduction and flattening of language to reflect the new cross-species commonality. The body became ‘the organism’, applicable to any sentient life form. The evolutionary physiologist G. H. Lewes attacked the ‘great ambiguities’ of the language used in debates about the mind and consciousness, and attributed most disputes to ‘language errors’ (Lewes, 1877: 156–7). He described consciousness variously as ‘a function of the organism’, ‘the transformation of a stimulus into a movement of self-conservation’, and a synonym for sensibility (Lewes, 1874: 110; 1877: 157; 1879a: 20). Romanes too downgraded consciousness: ‘The genesis of self-consciousness marks a comparatively low level in the evolution of the human mind’ (Romanes, 1888: 233). Reason, hitherto so prominent as a unique human feature in the work of the speculative philosophers, who were referred to derisively as ‘metaphysicians’ (Lewes, 1879b: 12), was similarly reduced. It became ‘ratiocination or comparison of ratios’, a basic mental formula for decision-making, or simply ‘the faculty of inferring’ (Romanes, 1888: 12). More often, ‘reason’ was replaced by ‘intelligence’, itself downgraded to mean an organism’s response to external stimuli and its capacity to adapt to changing circumstances, a ‘function of sensibility’ (Lewes, 1879b: 124). It was as evident in an earthworm responding to leaves blocking its burrow, Romanes suggested to Darwin, as in a human (Smith, 2013: 110). Thought was largely a function of sentience, ‘a logical combination of feelings’, as Lewes described it (Lewes, 1877: 163).

The purpose of this shift to reductive language was threefold. First, language applicable across species accentuated similarities, excised difference, and implied common origin. It supported the argument of Romanes that there was a ‘very strong *prima facie* case in favour of the view that there has been no interruption of the developmental process in the course of psychological history.…The mind of man, like the mind of animals…has been evolved’. His books set out to show further that human intelligence had evolved *from* animal intelligence, ‘by comparing the one with the other, in order to ascertain the parts where they agree, and the points where they differ’ (Romanes, 1888: 6–7).

Second, the new shared language allowed faculties that had hitherto been seen as the unique domain of the human to be attributed to all, or at least most, species. If reason was simply a matter of ratiocinative deductions and inferences, then it could equally be a function of the animal or human organism. Even the ability to abstract, a cornerstone of the Lockean concept of human mind, was within animal capacity, hidden from us only by their lack of language. Non-human animals could, Romanes claimed, take in and retain together several combinations of simple ideas and recognise choices of things, their only problem being that they could not express this verbally. The capacity for language, however, was not a sine qua non for the capacity to abstract: ‘The difference between man and brute in abstraction is merely terminology’ (Romanes, 1888: 32–3).

Third, with the consciousness gap reduced and mental faculties shared across species, not only could the evolutionary gap between humans and animals be seen as closely graduated, but it could even overlap. Romanes described a scale of articulation beginning at meaningless imitation and culminating in intentional attribution of meaning attached to words. The higher animals stood somewhere in the midpoint of the scale, where they understood the signification of articulate sounds or words, rather than simply tone or gesture. A dog, for example, could understand the word ‘whip’, thereby showing more intelligence than if it merely understood the gesture of whipping or the sight of an actual whip. Some human idiots, however, occupied the lowest, purely imitative, point of the scale, along with talking birds, young children, and some savages. Other idiots (along with deaf mutes and young children) occupied the next point, meaningless but spontaneous articulation, something Romanes claimed to have frequently witnessed for himself. Yet another class of idiot, too low in the scale themselves to speak, attained the same position as higher animals, understanding words independently of the objects they described, although unable to articulate themselves. On the evolutionary scale, therefore, not only were some animals very close to the conscious ability levels of some types of human, but in certain cases they actually exceeded them (Romanes, 1888: 121–3). Humans could no longer lay claim to a realm of competence unattainable by the animal, when the idiot part of their species lagged behind the dog and the ape.

This contraction, consolidation, and flattening of language had the effect of displacing the idiot from an already precarious niche at the bottom of the human scale in Victorian culture to an even more liminal position somewhere among or even below the higher animals. The idiot had for some time occupied a position that placed them alongside animals in the 19th-century imagination. Wordsworth’s burbling idiot boy was less intelligent than his horse; Dicken’s idiot ‘hero’ Barnaby Rudge communicated and shared understanding with his pet Raven Grip far better than he could manage any human relationship; while in *Nicholas Nickleby*, the imbecile Smike was a dog-like youth showing the canine qualities of blind affection and loyalty to Nicholas (Dickens, 1839, 1841; Wordsworth, 1998[1800]). The difference was that the new comparative psychology appropriated this cultural formulation, transformed it into an explanatory proof of evolution, and offered it back bearing a scientific authority it had hitherto lacked. The insertion of a scientifically fashioned idiot mind into the evolution debate was a critical component in the psychological refashioning of human and animal identity, and the production of a new materialist notion of consciousness residing in, and dependent on the quality of, the brain organ.

## The anthropomorphism debate in the new psychology

There was criticism of the tendency towards anthropomorphism of the most eager Darwinians, particularly Romanes, and their attribution of conscious intention to animal action. Notable critics were G. H. Lewes (preceding Romanes’ work), George Mivart, and C Lloyd Morgan. However, the parameters set by Romanes for comparative psychology endured until at least the end of the century largely intact and accepted, even by his critics. His consistent argument that, however large the gap might appear at times, the difference between the human and the animal was one of degree, not of kind, was resilient and underpinned work across comparative psychology.

Lewes, an enthusiastic evolutionist, nevertheless found himself ‘constantly thwarted by the fallacies of anthropomorphic interpretation’, and attacked the claims of ‘the various eminent writers who have attempted an Animal Psychology’ for their failure to note the conspicuous differences in the mental states of man and animal (Lewes, 1879b: 122–3). He famously declared, ‘The animal feels the Cosmos, and adapts himself to it. Man feels the Cosmos, but he also *thinks* it; again he feels the social world, and thinks it’ (Lewes, 1874: 124). The critical difference between man and animal, Lewes argued, was man the social being: ‘Man is not simply an animal organism, he is also a unit in a social organism’ (ibid.: 109). Nevertheless, he agreed that human intellect emerged from animal intelligence and that both intellect and conscience were products of animal impulses, which blended into human emotions. Consciousness, he maintained, could exist in different forms. Insects, crustaceans, and molluscs have sensibility, their actions not a reflexive function of an unfeeling nervous system but a form of sentient consciousness: ‘We shall do well to hold fast by the maxim that to have a sensation, and to be conscious of it, are one and the same thing’ (Lewes, 1860: 43–4). The difference between the lowest and the highest form of life was not that one lacked consciousness while the other had it; it was that man could attend to and direct consciousness in a way that lower forms of life could not (ibid.: 53). Lewes’ anti-anthropomorphism distanced him from some of the conclusions Romanes would reach in the following decade, but his framing of the animal–human continuum occupied a large tract of common ground.

The zoologist and comparative anatomist George Mivart launched an attack on Darwinian anthropomorphism in his 1876 work *Lessons From Nature*, denouncing the trend ‘to exaggerate the emotions of brutes, give them an intellectual appearance…he [Darwin] makes first gregariousness and then social sympathy the origin of our moral perception’ (Mivart, 1876: 208). Mivart denied the potential for morality or any recognisable form of reason in non-human animals, seeing them as uniquely human faculties. Such was the exaggeration of animal intelligence, he argued, that a book needed to be written on animal stupidity, citing dogs that attack their own masters when attacking their enemies and mating birds who fail to recognise each other (ibid.: 241–2). He denied neither sentience nor feeling to animals, but saw their highest activities, those that could be termed sensitive or emotional, as largely instinctive (ibid.: 192). He defined humanness as the possession of self-consciousness, intellectual memory, reason, will, and language. In his rhetorical question to himself about whether these truly were universal human attributes, he found one exception: ‘Are such powers, however, possessed by all mankind? Putting aside idiots as beings whose latent faculties are inaccessible and who are manifestly in an abnormal pathological condition, we have no hesitation…in affirming that they are’ (ibid.: 198). Mivart frequently disputed with Romanes during the 1880s over his ceding of large areas of the animal–human borderland in the quest to prove mental evolution and close the consciousness gap. However, even Mivart left one human grouping firmly on the wrong side of the border. Animal and human species did overlap, and it was the idiot, instinctive, irrational, reflexive, and partially conscious, who occupied the same stupefied mental state as Mivart’s animals. This non-humanness in human form was, for Mivart, a signifier of the complex perfection and uniqueness of the fully formed human as opposed to less conscious, more instinctive forms of life.

Also militating to some extent against Romanes’ anthropomorphic interpretations of animal actions was his friend and fellow comparative psychologist C. Lloyd Morgan, author of a series of major works, including *Animal Life and Intelligence* (1890–1), *The Springs of Conduct* (1892), *Habit and Instinct* (1896), and *Animal Behaviour* (1900). Lloyd Morgan is remembered most famously for his ‘canon’, which called for scientific rigour in the study of animal mental states:

In no case is an animal activity to be interpreted as the outcome of the exercise of a higher psychical faculty, if it can be fairly interpreted as the outcome of the exercise of one which stands lower in the psychological scale. (Morgan, 1894)

His point was clear. Comparative psychologists could not ‘know’ the mental states of animals; they could only observe animal behaviours and infer the mental attributes that caused them. They should not, therefore, attribute humanlike motivations for behaviours if instinct or reflex could equally well explain what the animal had done. Only when the animal showed conscious intelligence by responding adaptively to previously unencountered stimuli could conscious intention be proposed as the explanation. Lloyd Morgan’s canon has been presented as an intellectual attack on Romanes’ anthropomorphism, and Morgan certainly took issue with Romanes on many of his interpretations or speculations, an example of which was an acerbic exchange on instinct in *Nature* in 1884 (Morgan, 1884a, 1884b; Romanes, 1884). However, as Costall has argued, the canon has been over-interpreted by both Morgan’s contemporaries and subsequent writers on animal behaviour as a ‘revolt’ against the idea that animals could have conscious intention at all. Morgan’s point, Costall suggests, was that ‘there were good evolutionary reasons for expecting to find different grades of mentality across different species, and the theory of evolution depended on such differences’ (Costall, 1993: 117). His canon was more a methodological caution than a sweeping denunciation of anthropomorphism and the premises of comparative psychology. The common ground between Romanes and Morgan was extensive, and Morgan laid out the reflex–instinct–conscious action mental evolution pathway with great clarity (Morgan, 1884a: 373), never denying the common origin of animal and human intelligence. His estimation of the consciousness gap was more extensive than Romanes’, and his interpretations of behaviours more sceptical. Nevertheless, the pair operated within a shared conceptual framework.

However much Morgan, Mivart, and others might appeal for scientific objectivity and a curbing of the anthropomorphism that infused Romanes’ work, the public showed an unquenchable appetite for instances of animals displaying human attributes. In 1879, the medico-psychologist Henry Maudsley felt obliged to take to the pages of *Mind* to debunk stories that a cat had committed suicide by strangling itself in the fork of a tree after its kittens had been drowned, and that a dog had deliberately drowned itself in a river after being moved to a new house. Both were accidents, rather than ‘distinctly conceived and deliberately perpetrated’ acts, he insisted, adding that we were still awaiting evidence of such a level of intentionality from any animal below man (Maudsley, 1879). Romanes was more disposed to give credence to anecdotes sent in by readers or reported in newspapers of animals displaying remarkably human behaviours. In *Animal Intelligence*, he recorded the alleged tendency of scorpions to commit suicide (by stinging themselves) when surrounded by fire, inseparable swans, pining love birds, ostriches prepared to die for love, Swiss cows displaying pride when awarded garlands at show days (and depression when failing to win a prize), and pigs opening latches and gates (Romanes, 1886[1882]: 222, 270–1, 334, 339). Reporting a story from *The Times* about a grief-stricken boa constrictor who had crawled upstairs and lain down to die in mourning beside his dead master, Romanes said, ‘We are left to speculate whether the double seizure of the man and the snake was a mere coincidence or whether the sight of its stricken master…precipitated its death’ (ibid.: 261). Lloyd Morgan refused to speculate, and in response to Romanes’ musings about scorpions conducted a series of gruesome experiments. Scorpions were slowly heated up to unbearable temperatures, burnt, drowned, and attached to burning phosphorous. At no point, reported Lloyd Morgan, while acknowledging the ‘barbarism’ of his own experiments, did any scorpion seek to end its misery by stinging itself to death (Morgan, 1883b). Readers responded by denouncing Lloyd Morgan for ‘unnecessary cruelty’. He was forced to defend himself by arguing that the infliction of pain and the death toll (around 60 scorpions) were justified in order to respond scientifically to what was ‘left as an open question by Romanes’. Scorpion suicide, he added, if a fact, would be ‘one of the strongest individual cases against the theory of evolution by natural selection’ (Morgan, 1883a). The possibility of suicidal ideation in animals remains a preoccupation of animal historians. Echoing Romanes, and in line with contemporary cross-disciplinary interest in the capacity of non-human species for self-determination and conscious decision-making, historians suggest that self-injurious behaviours among humans and non-humans form a continuum. This underscores an argument that it is not only humans who are able to reflect upon life and death (Peña-Gúzman, 2017; Ramsden and Wilson, 2014).

As animals travelled upwards in human estimation in the final decades of the 19th century, seen increasingly as conscious, emotional, intentional, and capable, they might have glimpsed the idiot hurtling downwards in the opposite direction, a form of human only partially conscious (if at all), thoughtless, unfeeling, and lacking awareness, intent, and motivation. In 1904, Martin W. Barr, superintendent of the Pennsylvania Training School for Feeble-Minded Children and president of the American Association for the Study of Feeble-Mindedness, wrote that ‘the idiot intelligently sees nothing, feels nothing, hears nothing, does nothing’ (Barr, 1904: 2). His words were chosen carefully, and derived directly from psychology. ‘Intelligence’ in this sense was used in its reductive form indicating interaction with external stimuli. The idiot, in Barr’s presentation, lacked even the sensorium that Lewes saw as the basic spark of consciousness: the sensory pathways to experience sight and hearing, the ability to feel, and in return to react; to interact with the environment in the way that sentient organisms should. Somehow this lowest form of human was not even sentient. While debates raged about whether dogs and scorpions could be moved to commit suicide, snakes and cows feel love and pride, and mourning cats hang themselves, the proposition about human idiots was that they could feel nothing at all.

## Anthropological and neurophysiological responses to evolutionary thought

Doctors working in the field of idiocy (and lunacy) showed a keen interest in the work underway in both anthropology and psychology to assert the importance of idiotic arrested minds in the story of mental evolution. At least 27 medical superintendents and doctors joined the Anthropological Society of London from the 1860s onwards, their position in mental science placing them at an intersection that united the two disciplines of psychology and anthropology.^1^ The Anthropological Society, formed by its radically racist polygenist (multiple human origins) leadership as a breakaway from the more traditional and largely monogenist (common human origin) Ethnological Society in 1863, grew with ‘amazing rapidity’ in its first two years, gaining 500 new members and a Manchester branch (Stocking, 1987: 25–7, 248). Members of the Society were as interested in the study of intellectual deficit at home as in the study of other races abroad, and were keen to make comparisons and links in the quest for the key to human origin. Anthropological journals reported on Broca’s comparison of idiot and negro skulls in Paris, British-born microcephalics, a large idiotic family in Norfolk, and the educability of idiots (‘Anthropological News’, 1868; Atkinson, 1866; Gore, 1863; ‘The Proceedings of the Anthropological Society of Paris’, 1863). Carl Vogt’s *Lectures on Man* were published in English in 1864, edited and introduced by James Hunt, the Society’s president. Vogt placed the idiot at a halfway stage in the human–animal continuum between primates and the lowest form of human, by which he meant a female bushman. He ‘proved’ this by measuring the skulls of an ape, a microcephalous idiot, and a negro woman, pronouncing the idiot as having passed the simian stage, but nowhere near even the lowest human stage (Vogt, 1864: 170).

John Langdon Down, the medical superintendent of the Earlswood Asylum for Idiots, joined the Anthropological Society of London in 1864. The Earlswood Asylum, in Surrey, had opened in 1855 as the world’s first purpose-built institution for idiots, and Down had been medical superintendent since 1858. Down was ambitious and keen not to sink into professional obscurity under the weight of his administrative and medical duties tending his idiot charges in a rural backwater. He announced in an early annual report that he ‘would take advantage of the opportunity afforded by the large concentration of young idiots being brought together within the “spacious walls” to establish a classification of the pupils, based on the degree of their intelligence and capability of intelligence’.^2^ As he became an enthusiastic attendee at Society meetings, lectures, and discussions, Down imbibed the reversion and recapitulation theories of anthropology, and sought his own evidence of human mental evolution on the wards of his Surrey asylum. In 1867, his ‘Observation of an Ethnic Classification of Idiots’ was published in the *Journal of Mental Science*. He argued that, based on his observations, the idiots of Earlswood could be divided into the five great racial classifications of the world: Caucasian, Mongolian, Malay, Aztec (or American), and Ethiopian (or African). This was in spite of their being the offspring of exclusively Caucasian parentage: ‘A considerable portion can be fairly referred to one of the great divisions of the human family other than the class from which they have sprung’ (Down, 1867). His evidence, he argued, was his observations on the wards of the ‘prominent eyes, the puffy lips…retreating chin…[and] woolly hair’ of the Ethiopians, the prominent upper jaws and capacious mouths of the Malays (South Sea Islanders) and the deep-set eyes and slightly apish nose of the American. There was also the ‘great Mongolian family’ with their flat broad faces, oblique eyes, wrinkled foreheads and large thick lips (ibid.).^3^ His explanation was that the puzzling emergence of these alien ethnicities among the idiot offspring of Caucasian parents was the result of atavistic reversion, causing ancient, partially evolved racial types to emerge in a more advanced population. Evident in Down’s work was the role of the idiot as a snapshot of earlier, lower stages of mental evolution (the lowest form being his Africans and Native Americans, the Mongolians and Malays being somewhat more mentally advanced). Abnormal adults in superior races represented atavisms, ‘the spontaneous reappearance in adults of ancestral features that had disappeared in advanced lineages’ (Gould, 1990: 135–6). Down’s proposition united psychology, anthropology, and mental science in a reframing of the semi-evolved idiot mind that fitted comfortably into the post-*Origin* system of scientific truth.

There was a similar crossover in the investigation of mental evolution between psychology, mental science, and neurophysiology, most markedly in the medical research facilities established by James Crichton Browne (who had offered his observations on laughing idiots to Darwin) at the West Riding Asylum, where he was medical director from 1866 to 1875. Like Down, Browne was keen to avoid the potential obscurity of provincial asylum superintendency by insinuating himself into prevailing intellectual debates about human origin, as well as by presenting his institution as a centre for cutting-edge medical research. His approach was to add a pathology laboratory (claimed to be the first of its kind in a British asylum) complete with vivisection facilities and a supply of animals, from frogs to monkeys, to his institution. He also encouraged experiments involving humans (although not vivisection, of course) that sought to develop knowledge of the diseased and arrested brains of the idiot and lunatic inmates. The location of both human and animal brain research in a mental institution emphasised the continuities and similarities between animal and lower-human mental states, and supported the notion of the evolved material brain organ as the location of the mind, thought, and consciousness. Distinguished medical men, mostly from Edinburgh and London, descended on the facility to carry out research on the human brain, through comparative vivisection work on animals and observation and investigation of the idiot and lunatic human patients. Their findings were published in the highly influential *West Riding Lunatic Asylum Medical Reports* between 1871 and 1876.

There was an easy fit between evolutionary comparative psychology theories of the mind and advances in neuroscience that were unlocking the secrets of the human brain, and in both the idiot mind was key. The West Riding research focused on two related areas: the evolution of the brain and consciousness, and cerebral localisation. The evolutionary explanation of mental states depended on a material conception of the brain, and needed to bury once and for all notions of Cartesian dualism and a separate, innate consciousness. Unfolding work on the development of cerebral hemispheres over the century came to be seen as critical evidence of the emergence of consciousness, explaining why lower vertebrates and amphibians with simple nerve systems could operate in a reflexive and unthinking way, even continuing to react reflexively when their brains were removed, while the higher mammals controlled their reactions through varying levels of conscious thought and intentionality emanating from the cerebral hemispheres. Related work on cerebral localisation hardened the theory that different parts of the brain controlled all functions of the body (Young, 1970) – an idea resisted by, among others, G. H. Lewes, who continued to argue that the body contained influential nervous systems, particularly in the lower spine, that were independent of the brain organ (Lewes, 1860: 4).

David Ferrier’s animal brain experiments at West Riding in the 1870s were pivotal in the understanding of cerebral localisation. He carried out a rather gruesome series of experiments ranging from partial to full removal of brain material from frogs through to dogs and monkeys, giving a solid evidential base to localisation theory. He wrote these up in the West Riding reports, where he offered fulsome thanks to Crichton Browne ‘for kindly placing at my disposal the resources of the pathological laboratory of the West Riding Asylum, with a liberal supply of pigeons, fowls, guinea pigs, rabbits, cats and dogs for the purpose of my research’ (Ferrier, 1873: 30). The medical purpose of the experiments was to make possible identification and removal of brain tumours in humans. Once the functions of different parts of the brain were understood, a tumour’s location could be precisely identified by its effects on the rest of the body, and precise drilling into the skull could then take place to remove it.

Descriptions of the effects of removal of parts of animal brains on their behaviour and character brought out uncanny resemblances between the cerebrally reduced animal and the idiot human. Writing about the removal of the antero-frontal lobes from monkeys, Ferrier described the deterioration in their intelligence in terms familiar to anyone who would claim to have studied or observed the idiot:

The animals operated on were selected on account of their intelligent character. After the operation…they had undergone a considerable psychological alteration. Instead of, as before, being actively interested in their surroundings, and curiously prying into all that came within their field of observation, they remained apathetic or dull, or dozed off to sleep, responding only to the sensations or impressions of the moment, or varying their listlessness with restless and purposeless wanderings to and fro. While not actually deprived of intelligence, they had lost to all appearance the faculty of attention and intelligent observation. (Ferrier, 1878: 36–7)

Lethargy, incuriosity, reflexive rather than conscious reactions to surroundings and stimuli, inattention, and restless and purposeless wandering were all familiar tropes relating to the characteristics of idiocy. Through the insults to its brain, the higher animal had been reduced to the state of the lower human. Thus, in the West Riding menagerie the removal of brain in animals, and the absence of it in humans, refined understanding of the evolved organism in which all consciousness and thought resided, and how it had come to be itself.

Other articles in the *West Riding Asylum Reports* included analyses of brain weight variations in deceased idiots, the relationship between convolutions of the brain and intelligence, and electro-excitability in neural disease (Crockley and Clapham, 1873; Lowe, 1873; Turner, 1873). Animal experimentation and neurological investigation of the idiot mind came together just as easily in Crichton Browne’s West Riding Asylum in Yorkshire as anthropology and idiot observation came together in Dr Down’s idiot asylum. The idiot mind, and its perceived affinities with the animal mind, was a gift that evolutionists simply could not ignore.

## The moralisation of evolved consciousness

The notion of an evolved consciousness seated solely in the material organ of the brain presented a challenge to moral philosophy. If consciousness was imbued with organic origins and was no longer a mysterious, spiritual, God-given entity that maintained some sort of inexplicable and unobservable link with the material body, where did morality arise from? As Young has put it, ‘Moral responsibility no longer held a separate, divinely ordained basis in the freedom of the will’ (Young, 1985: 58). The choice for those who believed in a universal innate morality was either to resist evolutionary theory and hold fast to their original (often creationist) beliefs, or to find a way of moralising evolved consciousness. The moral philosopher and troubled Christian Henry Sidgwick (Lazari-Radek and Singer, 2014: 4–5) questioned whether the threat to ethics from evolutionary theory was as great as had been feared. In an article for *Mind* in 1876, he wrote,

The theory of evolution…has little or no bearing upon ethics. It is commonly supposed that it is of great importance in ethical controversy to prove that Moral Faculty is derivative and not original, and there can be little doubt that this conclusion follows from the theory which we are now considering. For when we trace back in thought the series of organisms of which man is the final result, we must – at some point or other, it matters not where – come to a living being, (whether called Man or not) devoid of moral consciousness, and between this point and that at which the moral faculty clearly presents itself, we must suppose a transition period in which the distinctly moral consciousness is gradually being derived and developed out of more primitive feelings and cognitions. (Sidgwick, 1876: 54)

A moral faculty, therefore, in Sidgwick’s view, simply evolved like other higher faculties of consciousness. It might not be present in primitive or less evolved minds, but there was no need to be concerned that it was something that developed rather than arriving fully formed: ‘Surely there can be no reason why we should single out for distrust the enunciations of the moral faculty merely because it is the outcome of a long process of development’ (Sidgwick, 1876: 54). The effect of such progression was to enable moral consciousness to be ‘gradually realised more and more’ (ibid.: 56). Sidgwick’s argument was that a moral being evolved through consciousness and, therefore, the more evolved the consciousness, the more moral the being. However, in evolutionary progress, *complexity* of organism was not *intrinsically* preferable or desirable, or a sign of a high level of moral evolution. An organism interacting with its environment in an extremely complex way could be a sign of defect or inadequacy. Sidgwick used the telling example of an ‘invalid’:

The responsiveness of an invalid’s organism to surrounding changes is often more discriminating than that of a man in strong health, though less effective for self-preservation. Indeed the common notion of ‘delicacy of organisation’ blends the attribute of subtle responsiveness to external changes with the very opposite of strong and stable vitality. (Sidgwick, 1876: 58)

Simple progression of an organism along the evolutionary path in adaptive response to its environment was not in itself a ‘good’. Sidgwick argued that complexity of organisation was preferable or desirable only if ‘as a means to some further end’. For the utilitarian Sidgwick, that further end was an increase in happiness, for the self and others. From this he derived his idea of ‘desirable consciousness’, which he equated with ‘ultimate good’. To be sentient was not a good in itself – the sentient being needed to have reached a level of morally evolved consciousness, which included an understanding of virtue and virtuous action. This was what made that consciousness desirable, and a means to happiness, which was in turn a part of ultimate good (Sidgwick, 1907[1874]). This moral faculty, Sidgwick argued, was a function of reason – which he termed ‘moral cognition’ – not instinct, and consequently was present only in a highly evolved consciousness (Sidgwick, 1876: 66; 1907[1874]: 34). It was in this way that morality evolved, as a progressive increase in enlightenment and moral sympathy in the minds of humans, even average humans – faculties hitherto lacking in the ‘rude’, defective, or unintelligent:

We may trace not only change in the moral code handed down from age to age, but progress in the direction of a closer approximation to a perfectly enlightened Utilitarianism.…We must distinctly notice another important factor…: the extension…of the capacity for sympathy in an average member of the community. The imperfection of earlier moral codes is at least as much due to defectiveness of sympathy as of intelligence; often, no doubt, the ruder man did not perceive the effect of his conduct on others; but often, again, he perceived them more or less, but felt little or no concern about them. (Sidgwick, 1907[1874]: 455)

Good was achieved, then, through this moral evolution, and the continuous ascent to desirable consciousness. The end of life was not simply to go on: it was to go on in a desirable way towards the ultimate good. The invalid or defective consciousness might be finely attuned to its environment, but this was an instinctive rather than a conscious interconnectedness, lacking reason or moral cognition, simply the self-perpetuation of an organism, perpetuating not pleasure, but pain, or the wrong sort of pleasure.

The work of Henry Maudsley, ‘the dominant medico-psychological specialist of the last third of the [19th] century’ (Scull, MacKenzie, and Hervey, 1996: 226), was influenced by Sidgwick’s account of the development of the moral faculty. Maudsley sought a neuropsychological explanation for its evolution, and placed a particular category of Sidgwick’s ‘invalid’ at its heart: the idiot and imbecile mind. In *Body and Will* (1883), a key chapter was titled ‘Congenital Deficience or Absence of Moral Feeling and Will’ (Maudsley, 1883: 243). His subject was ‘the function of will in the highest moral sphere – the region of moral feeling which, representing the highest reach of evolution, is the highest consummate infloresence of human culture’ (ibid.). To demonstrate the progress of the moral faculty, he began with congenital idiots, who ‘disinherited of their human birthright by reason of natural defect of bodily structure generally and of cerebral structure and function in particular, are incapable of a normal mental development, and some of them incapable of any moral development whatsoever’ (ibid.: 244). These were ‘human beings…so enthralled somehow in the meshes of insatiable matter, that they are without the potentiality of becoming truly human…a *reductio ad absurdum* of humanity’ (ibid.). Maudsley accepted the consensus that consciousness evolved from reflex through instinct to conscious action, hence his assertion that the congenital idiot brain was somehow enmeshed in reflex materiality with no conscious being.

He then proceeded from the Sidgwickian premise that consciousness was not in itself intrinsically desirable or of any utility apart from the amoral self-perpetuation of the organism. In Maudsley’s system, after achieving consciousness, the mind then had two further planes to which it needed to ascend. The first of these was conscious intelligence; the second was moral reasoning. Some minds could ascend to the first plane, and achieve the vestiges of intelligence, but not reach the second. They could therefore be intelligent without being moral. Such beings, whom Maudsley referred to using the neuter pronoun, were ‘not notably deficient in intelligence [and] can learn quickly when it pleases particularly in special lines of knowledge for which it shows a singular talent’ (Maudsley, 1883: 246). Such an amoral human would show acute cunning ‘to gratify its evil inclinations…a moral idiot without being an idiot in self-seeking and self-serving intelligence’ (ibid.). Maudsley was presenting, with the scientific and medical authority of the mental scientist, a Sidgwickian ‘invalid’, an organism interacting intelligently with its environment but without desirable consciousness. As he summarised, ‘The defect of intelligence is that it is capable of only half its function, being acute to apprehend self, impotent to apprehend the social not-self’ (ibid.).

The moral idiot, later to become the moral imbecile, thus conceptualised by Maudsley was a dangerous being, a monstrous, asocial Sidgwickian human organism intelligent enough to seek its own ends but, in the absence of the moral reasoning that formed desirable consciousness, oblivious and icily indifferent to the feelings of others. The amorality was more often than not invisible, masked by the intelligence, a mind that knew right from wrong but cared nothing about it:

The creature is truly an asocial being. So incorrigibly vicious as it is…so perserveringly set on evil doing, so utterly incapable of penitence, everybody who has to do with it feels in the end it is not really responsible for its conduct, perceives sadly that the severest punishment cannot do it the least good, and is constrained to acknowledge that it labours under a natural incapacity of moral development: it is congenitally conscienceless. (Maudsley, 1883: 246)

Maudsley’s moral imbecile was a human arrested in their mental evolution, at an animalistic, amoral stage beyond the material, reflexive, congenital idiot. Such a being was a threat, a semi-evolved egoistic brain that could plan and cause harm but cared nothing for the effects of its actions. A professional medical eye was needed to detect them, beneath their disguising veneer of normality. The medical eye did its work, and the moral imbecile was a major target of the 1913 Mental Deficiency Act, numbered among the 133,000 feeble-minded identified in the 1901 census, destined for either lifelong incarceration in mental deficiency colonies or a lifetime of community supervision (Thomson, 1998).

It is notable that although Maudsley is famed as a degenerationist and eugenicist, the line of thinking that led to his conception of the moral imbecile was far from eugenic. He gave a eugenic tinge to his conclusions by claiming that as the moral sense was the highest reach of evolution, it was the most recently developed of the faculties of mind and therefore most prone to instability and dissolution (Maudsley, 1883: 243). His thinking, however, derived from the comparative psychologists such as Romanes, who mapped out the evolutionary journey of the mind using the arrested state of idiocy as a key linking point between the animal and human mental states. This was not a degenerative journey backwards, but a progressive journey during which a section of the species had alighted early and remained in the company of other, lower, species, reflexive and instinctive, virtually devoid of consciousness. It also arose from the utilitarian philosophical response, led by Sidgwick, to the challenge that mental evolution posed to notions of morality. Maudsley’s moral imbecile gave medical form and explanation to the undesirable consciousness of Sidgwick’s invalid type, a finely tuned organism interacting amorally with its stimuli, relentlessly pursuing the pleasures of asocial self-perpetuation. Just as Ferrier’s monkeys, devoid of their antero-frontal lobes, became removed from the social world, incurious and incapable of learning, so the idiot, and the closely related imbecile, operated in an amoral closed feedback loop that merely perpetuated a painful existence for its own sake.

## Conclusion

In these ways, across the disciplines of comparative psychology, anthropology, neuroscience, philosophy, and psychiatry, a new framing of the idiot mind occurred in the last decades of the 19th century. This framing was a fabrication, built from anecdote, superficial observation, long-standing tropes, assumption, and a quest to describe a consciousness-free, or almost consciousness-free, form of human mind to demonstrate the truth claims of mental evolution. This version of the idiot mind has been used as an explanatory artifice, dressed as scientific fact, to prove consciousness theory. To borrow from Mitchell and Snyder’s (2000) concept of the narrative prosthesis in literature, the disabled brain has been a useful crutch to carry the arguments of evolutionary psychologists to their conclusions. The reframing of the idiot mind was accompanied by a reframing of the animal mind in comparative psychology that inflated the consciousness claims related to non-human animals. This development was led by the overtly anthropomorphic Romanes, but its underlying assumptions were shared by the more measured and ‘scientific’ C. Lloyd Morgan. These new conceptions of human idiot and animal mental states allowed minds across species not only to come close to convergence, but even to swap positions at a certain nexus on the evolutionary scale of development. All of this provided a neat proof of evolutionary theory.

The idiot mind came to be seen as a fascinating living reversionary snapshot of a number of mental states – embryonic, early, pre-human, reflexive, instinctive, pre-conscious, animalistic, and amoral among them. Where difficult gaps existed in the evolutionary story, the infinitely malleable idea of the empty intellectually disabled mind could be relied upon to fill them – the gap between lower human and animal, between higher and lower animal, between desirable and undesirable consciousness, between the moral and the amoral being. Psychology, neurophysiology, and mental science gave scientific authority and substance to the philosopher Sidgwick’s refashioning of moral cognition in response to the challenge of evolutionary theory.

The role of the idiot mind as a critical intermediary in this process of ‘biologising of human behaviour’ (Sperling, 1988: 189) has enjoyed a lengthy if largely unacknowledged and unnoticed shelf life. In 1978, the biologist E. O. Wilson wrote in ‘On Human Nature’ that the ‘mentally retarded’ were living analogues of a simpler state, who

represent a sudden and dramatic step downward in ability.…Their exchanges with others entail little that can be labelled as truly human communication. Cultural behaviour thus seems to be a psychological whole invested in the brain or denied it in a single giant step, yet the non-cultural retardates retain a large repertory of more ‘instinctive’ behaviour the individual actions of which are complex and recognisably mammalian. (Wilson, quoted in Sperling, 1988: 189)

The observational anecdotalism of the early comparative psychologists, who sought to solve the evolutionary consciousness puzzle by reducing the scope of human capacity in contradistinction to an amplified version of animal consciousness, has enjoyed a revival since the latter part of the 20th century. This has been in part a reaction against behaviourism’s domination of mid-20th-century psychology, with its crude rejection of any possible epistemology of internal mental states. Bernard Rollin has rehabilitated Romanes from ‘simplistic purveyor of anecdote’ to conceptually astute and subtle thinker (Rollin, 1989: 46). Harriet Ritvo has noted that the kind of information that was originally dismissed as anecdotalism has re-emerged in a more respectable form as ethology (Ritvo, 2000: 852).

As Kimler has commented on the challenge presented by the need to account for the evolution of consciousness, ‘The science cannot evade the vexing issue of how to demonstrate the unobservable’ (Kimler, 2000: 860). The dramatic contrast between humans and other species has always presented a challenge to evolutionary theory, and has spawned two enduring factional positions, one proposing human uniqueness and the other seeking to demonstrate that ‘the claimed differences between them and other species must be somehow illusory, or of negligible importance compared with the similarities’ (Bickerton, 2000: 862). It is consequently no surprise that observational anecdotalism has regained respectability, and that the old trope of the semi-developed idiot brain has been recalled to perform some conceptual heavy lifting in the quest for the coveted grail of animal consciousness.

The significance of this intellectual current today is that its philosophical and moral speculations endure, sometimes in extreme form. At the heart of the ethical philosopher Peter Singer’s idea of ‘speciesism’ lie echoes of late-Victorian fabrications of consciousness. Just as these concepts of the idiot mind sought to embed mental evolution as a universal truth from the 1860s onwards, so they have been used to advance consciousness claims for animal species (and euthanasia claims against ‘retarded’ human infants) since the 1970s. As Singer writes,

Adult chimpanzees, dogs, pigs, and members of many other species far surpass the brain-damaged infant in their ability to relate to others, act independently, be self-aware, and any other capacity that could reasonably be said to give value to life. With the most intensive care possible, some severely retarded infants can never achieve the intelligence level of a dog. (Singer, 1975: 18)

The ethics of Singer, a utilitarian heavily influenced by Sidgwick, and who professes to ‘make the strongest case…for the views that Sidgwick defended’ (Lazari-Radek and Singer, 2014: xiii), have some roots in these late-Victorian concepts of idiocy. These ideas therefore informed both the making of the Mental Deficiency Act at the beginning of the 20th century, and the theoretical assumptions of the animal liberation movement at the century’s end, as well as contributing conceptions of the intellectually disabled mind that endure in law, psychology, moral philosophy, and medical ethics. Their real-life implications have undoubtedly been substantial and significant. It is important, therefore, that the role of these ideas of idiocy as an enduring explanatory artifice, an intellectual crutch, in the account of mental evolution and the emergence of consciousness is understood and acknowledged.

## Notes

Thanks to Hallvard Lillehammer, Daniel Pick, Joanna Bourke, Sarah Marks, Sam Ashenden, Jason Edwards, and C. F. Goodey for their thoughtful comments on drafts of this work. Thanks also to the anonymous reviewers who helped to improve the text.

1. Royal Anthropological Institute, *Fellows Database*, 2015.

2. Earlswood Annual Report, 1859, Surrey Records Office (SRO), 392/1/1.

3. This was of course the origin of Down’s ‘discovery’ of Mongolian imbecility, later to be renamed Down’s syndrome. His other ethnic classifications, and the racial science he used to form them, have been largely, and quietly, forgotten.
